# Multi-omics Quality Assessment in Personalized Medicine Through European Infrastructure for Translational Medicine (EATRIS): An Overview

**DOI:** 10.1007/s43657-024-00170-0

**Published:** 2025-04-01

**Authors:** EATRIS-Plus Multi-omics working group and Stakeholders, Patricia Alonso-Andrés, Davide Baldazzi, Qiaochu Chen, Elisa Conde Moreno, Lorena Crespo-Toro, Kati Donner, Petr Džubák, Sara Ekberg, Maria Laura García Bermejo, Daniela Gasparotto, Bishwa Ghimire, Jolein Gloerich, Alain van Gool, Janine Habier, Marián Hajdúch, Rashi Halder, Sari Hannula, Peter-Bram ´t Hoen, Hanna Lindgren, Yaqing Liu, Roberta Maestro, Tom Martin, Pirkko Mattila, Lukáš Najdekr, Kenneth Nazir, Anna Niehues, Anni I. Nieminen, Jessica Nordlund, Emanuela Oldoni, Elin Övernäs, Aino Palva, Maija Puhka, Ileana Quintero, Miren Edurne Ramos-Muñoz, Esperanza Macarena Rodríguez-Serrano, Sabrina Saracino, Andreas Scherer, Leming Shi, Jarmila Stanková, Tanushree Tunstall, Beatrice Valenti, Janneke Weiss, Bhagwan Yadav, Yuanting Zheng, Patricia Žižkovičová

**Affiliations:** https://www.eatris.eu

**Keywords:** Multi-omics, Quality, Reference samples, European infrastructure for translational medicine, Multi-omics toolbox

## Abstract

Molecular characterization of a biological sample, e.g., with omics approaches, is fundamental for the development and implementation of personalized and precision medicine approaches. In this context, quality assessment is one of the most critical aspects. Accurate performance and interpretation of omics techniques is based on consensus, harmonization, and standardization of protocols, procedures, data analysis and reference values and materials. EATRIS, the European Infrastructure for Translational Medicine (www.EATRIS.eu), brings together resources and services to support researchers in developing their biomedical discoveries into novel translational tools and interventions for better health outcomes. Here we describe the efforts within the Horizon 2020 EATRIS-Plus project and activities of member facilities of EATRIS towards quality assessment of pre-clinical sample processing, clinical omics data generation, multi-omics data integration, and dissemination of the resources in a Multi-Omics Toolbox, which is the principal deliverable of the EATRIS-Plus project for the consolidation of EATRIS towards translational medicine.

**Supplementary Information:**

The online version contains supplementary material available at 10.1007/s43657-024-00170-0.

## Introduction

Precision medicine relies on sensitive and specific detection of biological variables that may support diagnosis, prognosis, and prediction of therapy response. In this context, omics technologies, which provide qualitative and quantitative information on thousands of molecular entities such as nucleic acids, proteins, and metabolites in a given biological sample, have proven invaluable tools. Moreover, two or more omics technologies may be integrated into multi-omics approaches, thus increasing the possibility of detection of molecular signatures, and providing a more comprehensive view of the molecular portrait and biomarkers of phenotype and the health status of an individual. Clearly, the accuracy of such a type of molecular diagnosis demands a high quality of data. This is becoming increasingly crucial, given the widespread use of omics approaches in routine diagnostics and the progressive expansion in the clinical applications. In addition to making data findable, accessible, interoperable, and reusable (FAIR-principles, https://www.go-fair.org/fair-principles) (van der Velde et al. 2022; Wilkinson et al. 2016), assuring a minimal level of data quality is essential for further use towards the benefit of individuals in healthcare and clinical care.

**Fig. 1:**
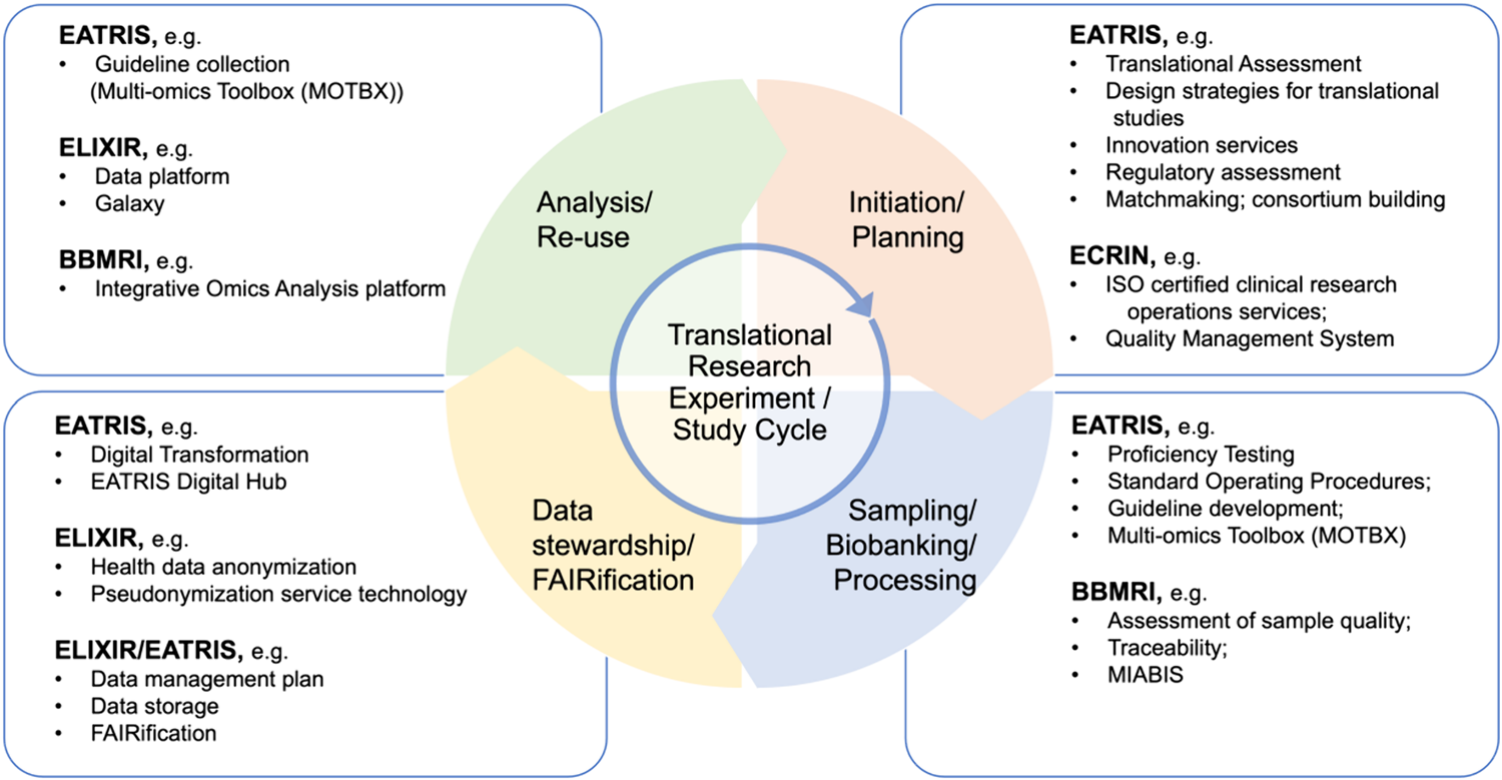
The translational biomedical process and involvement of selected biomedical Research Infrastructures (RIs) as stakeholders in quality assessment. EATRIS is involved in quality assessment checkpoints along the translational trajectory, while other RIs tend to focus on specific aspects or technologies. ECRIN, https://ecrin.org; BBMRI-ERIC, https://bbmri-eric.eu; Elixir, https://elixir-europe.org

Integrated multi-omics data analysis and interpretation require careful design of experiments and associated data analysis procedures to enable optimal use of research resources. Importantly, quality needs to be assessed and maintained throughout the whole process of generation and analysis of multi-omics approaches. In this regard, several initiatives have been undertaken to promote best practices, ranging from a proper definition of the experimental question and study design, sample handling, data analysis and stewardship, and re-use of approaches and data. The exceptional structure of European Infrastructure for Translational Medicine (EATRIS), which spans access to technologies, expertise, and services in most areas of biomedical research and thus overarches the process of personalized medicine, provides an integrative platform to prevent siloing, thereby ensuring services and quality assurance along the entire translational trajectory from conception and evaluation of a study to the data re-use (Fig. 1).

The EATRIS-Plus project was launched to deliver innovative scientific tools to support the long-term sustainability strategy of EATRIS as one of Europe’s key research infrastructures for Personalized Medicine. As part of the EATRIS-Plus project, a multi-omics dataset from about 100 healthy individuals was generated and compiled. The data of this pilot project are being made available for further analyses by stakeholders involved in biomedical research, healthcare, and drug development. The multi-omics study was based on samples collected from volunteers within the Czech Genome Project (https://czechgenome.iabio.eu/). Here we outline and discuss the efforts that the EATRIS-Plus initiative took to address comparative quality assessment (QA) of omics data generation and analysis. Procedures and results of this initiative have been compiled in the Multi-Omics Toolbox (MOTBX) (https://motbx.eatris.eu), (see below).

### Proficiency Testing for Assessing Pre-Analytical Sample Processing Methods

Proficiency Testing (PT) programs are external quality assessment (EQA) tools that aim to evaluate the performance of laboratories in conducting specific measurements or tests, ensuring ongoing quality monitoring, and promoting the standardization of procedures to achieve greater consistency and reproducibility in results (Miller et al. 2011).

Participation in PT programs enables laboratories to gauge the strengths and weaknesses of their procedures by comparing performance and reproducibility of their results with those of their peers and allowing to validate and enhance performance, identify potential issues in testing and processing or technical problems with equipment or reagents, compare and harmonize methods and procedures, assess precision and accuracy, evaluate operator capabilities, provide staff education, and instill confidence in laboratory staff and users (Analytical Method Committee 2010; Brookman et al. 2011). PT programs can also offer valuable insights into method-trueness, particularly in cases where reference materials are not available, thus supporting analytical method validation (Analytical Method Committee 2010).

PT programs play a crucial role in the quality management system of laboratories by ensuring consistent delivery of high-quality data and monitoring data reliability. Laboratories in which the results deviate significantly for the expected values (high z-scores) necessitate prompt corrective actions to be taken. To achieve this, implementing a comprehensive set of measures is essential. These measures encompass the utilization of standard operating procedures (SOPs) and validated protocols, incorporating internal quality controls such as reference materials and control charts, active participation in PT programs, and obtaining certification or accreditation according to recognized standards such as ISO15189/IEC, ISO/IEC 17025 and ISO 9001 (Meggendorfer et al. 2022). Indeed, it has been demonstrated that laboratories engaged in PT programs exhibit robust internal quality control procedures and consistently achieve better z-scores (Taverniers et al. 2004; Verderio et al. 2022).

**Fig. 2:**
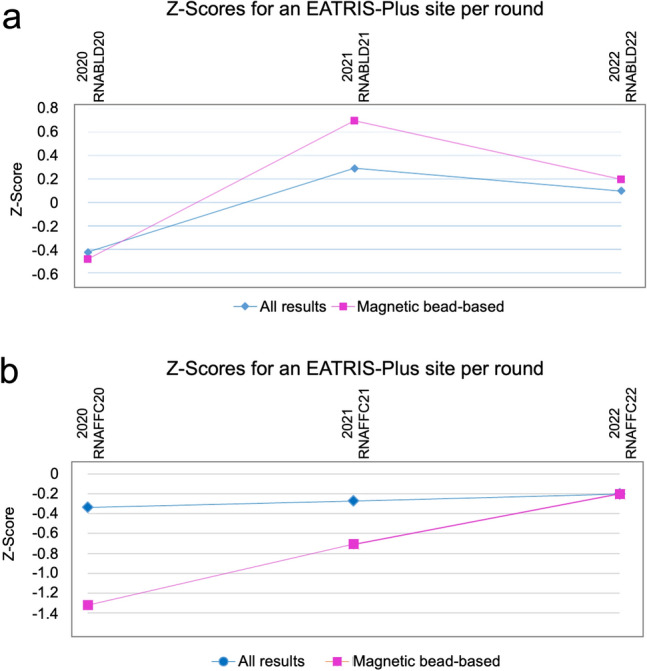
External quality assessment. History of z-Scores of one of the four EATRIS-Plus omics sites participating in the “RNA Extraction from Whole Blood” scheme (**a**), and “RNA Extraction from FFPE Tissue”-scheme of the IBBL PT program from 2020 until 2022 (**b**). In the long run, a large proportion of results giving rise to |z|> 2 (more than 5%) and |z|> 3 (more than 0.3%) indicates either a biased mean, or a standard deviation of the participant which is higher than the Proficiency Testing Standard Deviation. The participating site used a magnetic bead-based RNA isolation method, whereas the comparative score “All results” is an average z-Scores from all participants using magnetic bead-based, silica membrane-based or other RNA isolation methods. FFPE, formalin-fixed paraffin-embedded

In the context of EATRIS-Plus, a PT program for biospecimen processing has been established by the Integrated BioBank of Luxembourg (IBBL, https://www.lih.lu/en/translational-medicine/translational-medicine-operations-hub/integrated-biobank-of-luxembourg-ibbl/; https://biospecimenpt.ibbl.lu/) to ensure the fitness-for-purpose of samples for the down-stream omics analysis. From 2020 to 2022, two EATRIS-Plus partners involved in omics analyses were enrolled in 26 processing schemes. The aim was to compare the efficiency of the sample processing methods to ensure the validity of the result, monitor, and improve performance by identifying potential problems, and prove consistency of performance over time. The laboratory´s performance can be compared to the performance of other laboratories and used to adjust the quality of the outcome. Figure 2 shows the case of an exemplary EATRIS facility with sequential participation in PT programs. The laboratory in Fig. 2a was able to improve the overall quality of its performance from 2020 to 2022, just as the reference laboratories did. The laboratory results in Fig. 2b show an improvement of the performance from 2020 to 2022, obtaining the quality to the level of the reference laboratories. It has become clear that participation in PT programs can play an important role in gaining information on the sample processing quality. In case specific problems are pinpointed in any of the determined parameters, the participating laboratory can take corrective measures, and this can lead to significant improvement in the quality of the sample processing. Participation in PT schemes represents a critical step also for omics analyses to ensure accurate, reliable, and trustworthy data.

### Multi-omics Reference Materials for Quality Assessment: Commercial and Research Materials

The options for analysis of biological samples with omics platforms are numerous, and there is no undisputed or unchallenged “perfect” way. Largely depending on the research or clinical question and the available material, extraction methods may vary, and many technical and statistical approaches may be employed, sometimes leading to tremendous discrepancies in the results (Shi et al. 2010).

All facilities of the EATRIS-Plus project utilize high-quality reference material in the daily work routine for their respective omics analysis platforms to monitor changes of quality over time and from experiment to experiment (Table 1). Gradual deterioration or improvement of the data quality from the reference material as well as batch effects and other “unwanted noise” indicates that corrective measures are needed. In addition to these intra-lab QA, inter-laboratory QA can also be conducted. However, unless all labs use the exact same methodology (an ideal condition for direct lab-to-lab comparison), the comparative analysis usually relies on the identification of consensus features (e.g., concentration of analytes in a biofluid, detection of small variants in a DNA or specific transcripts in an RNA).

**Table 1:** Reference material for omics technologies Adapted from MOTBX website (https://motbx.eatris.eu)

Omics platform	Resource title	Resource description	Link to resource	Commercial/research samples
Genomics	NIST NA12878: whole genome sequencing	NA12878 is a human genomic DNA sample standardized by the National Institute of Standards and Technology (NIST). The donor subject, NA12878's mother, carries a single bp (G-to-A) transition at nucleotide 681 in exon 5 of the CYP2C19 gene (CYP2C19*2). This genetic change creates an aberrant splice site, resulting in a 40-bp deletion at the beginning of exon 5 (from bp 643 to bp 682)	https://catalog.coriell.org/0/Sections/Search/Sample_Detail.aspx?Ref=NA12878&Product=DNA	Commercial
Metabolomics	NIST sample SRM 1950	NIST SRM 1950 Metabolites in Human Plasma is intended to have metabolite concentrations that are representative of those found in adult human plasma. The plasma used in the preparation of SRM 1950 was collected from both male and female donors. This SRM was designed to apply broadly to the field, not toward specific applications. Therefore, concentrations of approximately 100 analytes, including amino acids, fatty acids, trace elements, vitamins, hormones, selenoproteins, clinical markers, and perfluorinated compounds (PFCs), were determined	https://www.ncbi.nlm.nih.gov/pmc/articles/PMC4823010/; https://www.sigmaaldrich.com/FI/en/product/sial/nist1950	Commercial
Methyl-seq	The SEQC2 epigenomics quality control (EpiQC) study: reference dataset	DNA methylation reference dataset for methylation profiling by high throughput sequencing (Methyl-seq). The full article can be found at 10.1186/s13059-021-02529-2	https://www.ncbi.nlm.nih.gov/geo/query/acc.cgi?acc=GSE186383	Research
Methyl-seq	DNA controls in EM-Seq Kit: unmethylated lambda and CpG-methylated pUC19	Unmethylated lambda DNA and CpG-methylated pUC19 DNA, two essential spike-ins employed in EM-seq library preparation. The unmethylated lambda DNA serves to evaluate conversion efficiency, while the CpG-methylated pUC19 DNA acts as a control to ensure accurate and reliable results. These spike-ins play a crucial role in validating the quality of EM-seq libraries, enabling researchers to achieve robust and precise outcomes in their experiments	https://international.neb.com/faqs/2019/06/04/what-levels-of-conversion-are-typical-with-the-control-dnas-supplied-in-the-em-seq-kit	Research
Multi-omics	Quality control and data integration of multi-omics profiling: Reference dataset	Multi-omics reference materials and practical tools to enhance the reproducibility and reliability of multi-omics reference material (Fudan Quartet samples) provided as part of the Quartet project. These well-characterized resources serve as quality control measures, ensuring accurate data integration in precision medicine studies	http://chinese-quartet.org/#/dashboard	Research
Proteomics	Peptide mix and software: promega catalog number V7491	The peptide mix and LC/MS instrument performance monitoring software includes a 6 × 5 LC–MS/MS peptide reference mix. This combination provides a comprehensive solution for monitoring and optimizing LC/MS instrument performance including sensitivity and dynamic range. Researchers can easily optimize their LC/MS system and ensure reliable and precise results	https://nld.promega.com/products/mass-spectrometry/mass-spec-reference-reagents/6-x-5-lc_ms_ms-peptide-reference-mix/	Commercial
Proteomics	A proteomics performance standard to support measurement quality in proteomics	National Institute of Standards and Technology (NIST) reference material (RM) 8323 yeast protein extract for benchmarking the preanalytical and analytical performance of proteomics-based experimental workflows	10.1002/pmic.201100522	Commercial
Proteomics	Characterization of a human liver reference material fit for proteomics applications	The National Institute of Standards and Technology (NIST) reference material RM 8461 human liver for proteomics for automated or manual sample preparation workflows, with the resulting data used to directly assess complete sample-to-data workflows and provide harmonization and benchmarking between laboratories and techniques	https://www.nature.com/articles/s41597-019-0336-7	Commercial
Proteomics	Evaluation of NCI-7 cell line panel as a reference material for clinical proteomics	A panel of seven cancer cell lines, NCI-7 cell line panel, as reference material for mass spectrometric analysis of human proteome. This publication shows that NCI-7 material is suitable for benchmarking laboratory sample preparation methods, but also NCI-7 sample generation is highly reproducible at both the global and phosphoprotein levels. In addition, the predicted genomic and experimental coverage of the NCI-7 proteome suggests the NCI-7 material may also have applications as a universal standard proteomic reference	10.1021/acs.jproteome.8b00165	Research
Transcriptomics	ERCC RNA spike-in mix: ThermoFisher Scientific catalog number 4456740	External RNA Controls Consortium (ERCC) RNA spike in mix consists of a set of 92 unlabeled, polyadenylated transcripts, derived and traceable from NIST-certified DNA plasmids, designed to be added to an RNA analysis experiment after sample isolation, to measure against defined performance criteria. Up until the design of such universally accepted controls, it has been difficult to execute a thorough investigation of fundamental analytical performance metrics. The transcripts are designed to be 250 to 2,000 nt in length, which mimic natural eukaryotic mRNAs	https://www.thermofisher.com/order/catalog/product/4456740	Commercial
Transcriptomics	Universal human reference RNA: ThermoFisher Scientific catalog number QS0639	The Universal Human Reference RNA is composed of total RNA from 10 human cell lines (Adenocarcinoma, mammary gland; Melanoma; Hepatoblastoma, liver; Liposarcoma; Adenocarcinoma, cervix; Histiocytic lymphoma, macrophage, histiocyte; Embryonal carcinoma, testis; Lymphoblastic leukemia, T lymphoblast; Glioblastoma, brain; Plasmacytoma; myeloma; B lymphocyte). Equal quantities of DNase-treated total RNA from each cell line were pooled to make the universal control	https://www.thermofisher.com/order/catalog/product/QS0639	Commercial
Transcriptomics	Total human brain reference RNA: ThermoFisher Scientific catalog number QS0611	Human brain total RNA is a total RNA sample extracted from neural tissue for use as a positive control in QuantiGene assays	https://www.thermofisher.com/order/catalog/product/QS0611	Commercial

In the case of multi-omics datasets management, batch detection and correction methods, and best practices for mitigation of unwanted noise are currently being developed (Goh et al. 2017; Ugidos et al. 2020).

While single omics reference materials are available and widely used as “ground truth” for the evaluation of the performance of the technologies and technology benchmarking (e.g. genomic DNA (Zook et al. 2019, 2020), tumor-normal paired DNA (Deveson et al. 2021; Fang et al. 2021; Jones et al. 2021), RNA (SEQC MAQC III Consortium 2014), protein (Friedman et al. 2011; Ivanov et al. 2013), and metabolite reference material (Ulmer et al. 2017)), multi-omics demands matched reference resources spanning DNA, RNA, proteins and metabolites. EATRIS-Plus members have used both commercial and research reference materials during the project.

### Commercial Omics Reference Material

To evaluate the process from acquisition of pre-extracted reference multi-omics material (RNA, DNA, metabolites, and proteins) to data generation, the EATRIS-Plus project employs both commercially available reference materials and research reference materials, namely the samples of the Fudan Quartet project (Table 1). Commercial quality references are regularly used by EATRIS-Plus sites, usually for longitudinal quality assessment. These reagents are processed according to the procedures used at each site and allow for a comparison of data quality between laboratories. However, for certain omics technologies commercial reference materials are available to check only specific technical steps but not the whole process (from sample preparation to data analysis). For example, no reference materials specifically designed for differential protein expression analysis are commercially available, and usually in-house generated standards obtained by mixing at defined ratios commercially available individual tryptic digests are used as a proxy.

### Research Omics Reference Material: The Fudan Quartet Project

**Fig. 3:**
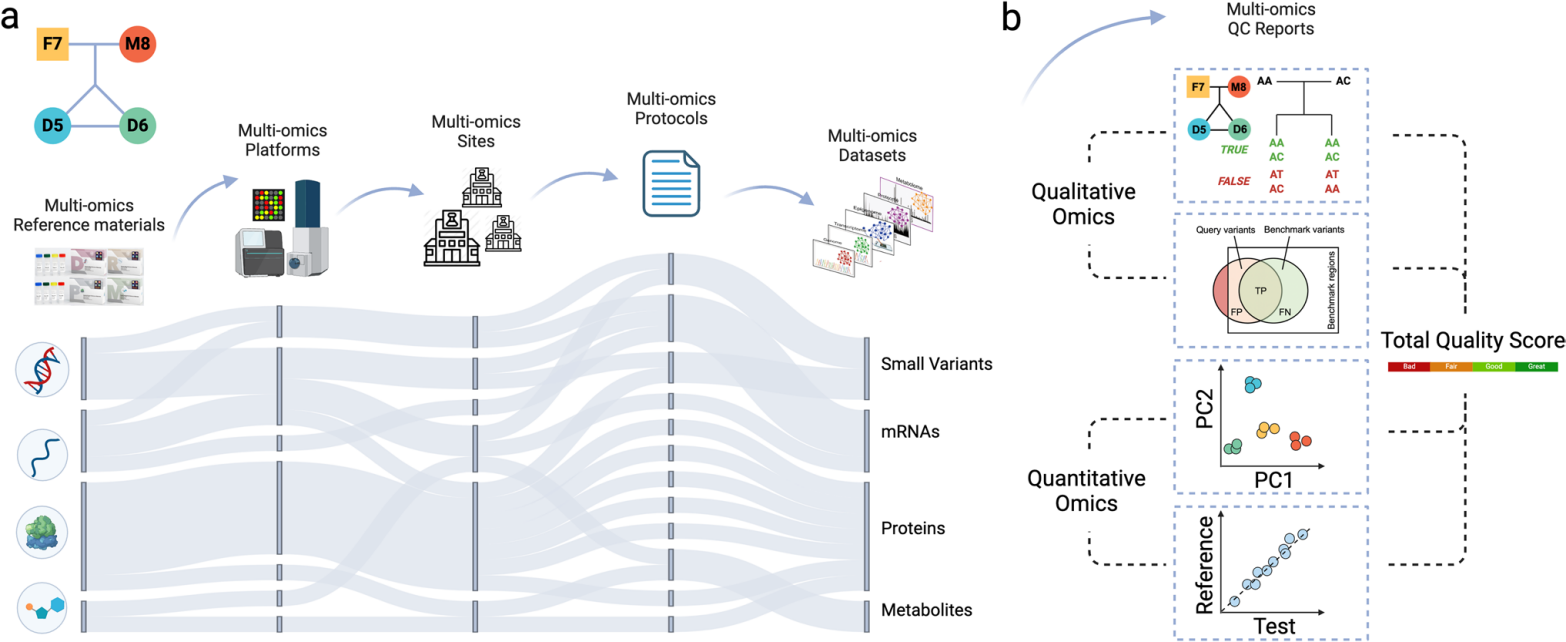
The Chinese Quartet reference materials and the quality assessment system. **a** Data generation by using Quartet reference materials across multiple platforms, sites, and protocols. **b** visual representation of several quality assessment parameters. The quality assessment system is embedded on the Quartet Data Portal (https://chinese-quartet.org/#/dashboard). A total quality score is calculated from several individual aspects of quality, for qualitative omics including Mendelian concordance rate and F1 score and for quantitative omics including signal-to-noise ratio (SNR) and relative correlation with reference dataset (RC) (Zheng et al. 2023). *QC* Quality control; *PC* principal component

In a PT effort aimed to assess the quality of the omics technology platforms of EATRIS-Plus partners, the EATRIS-Plus project acquired Quartet multi-omics reference materials from Fudan University (Shanghai, China). The Quartet reference materials encompass DNA, RNA, protein, and metabolites, all derived from B-lymphoblastoid cell lines obtained from a familial quartet consisting of parents and monozygotic twin daughters (https://chinese-quartet.org) (Yang et al. 2023; Yu et al. 2023a, 2023b; Zheng et al. 2023). The Quartet Project is a pivotal resource, providing “multi-omics ground truth”, best practices, and computational methods for objective assessment of proficiency and reliability of data generation processes in participating laboratories (Fig. 3).

**Fig. 4:**
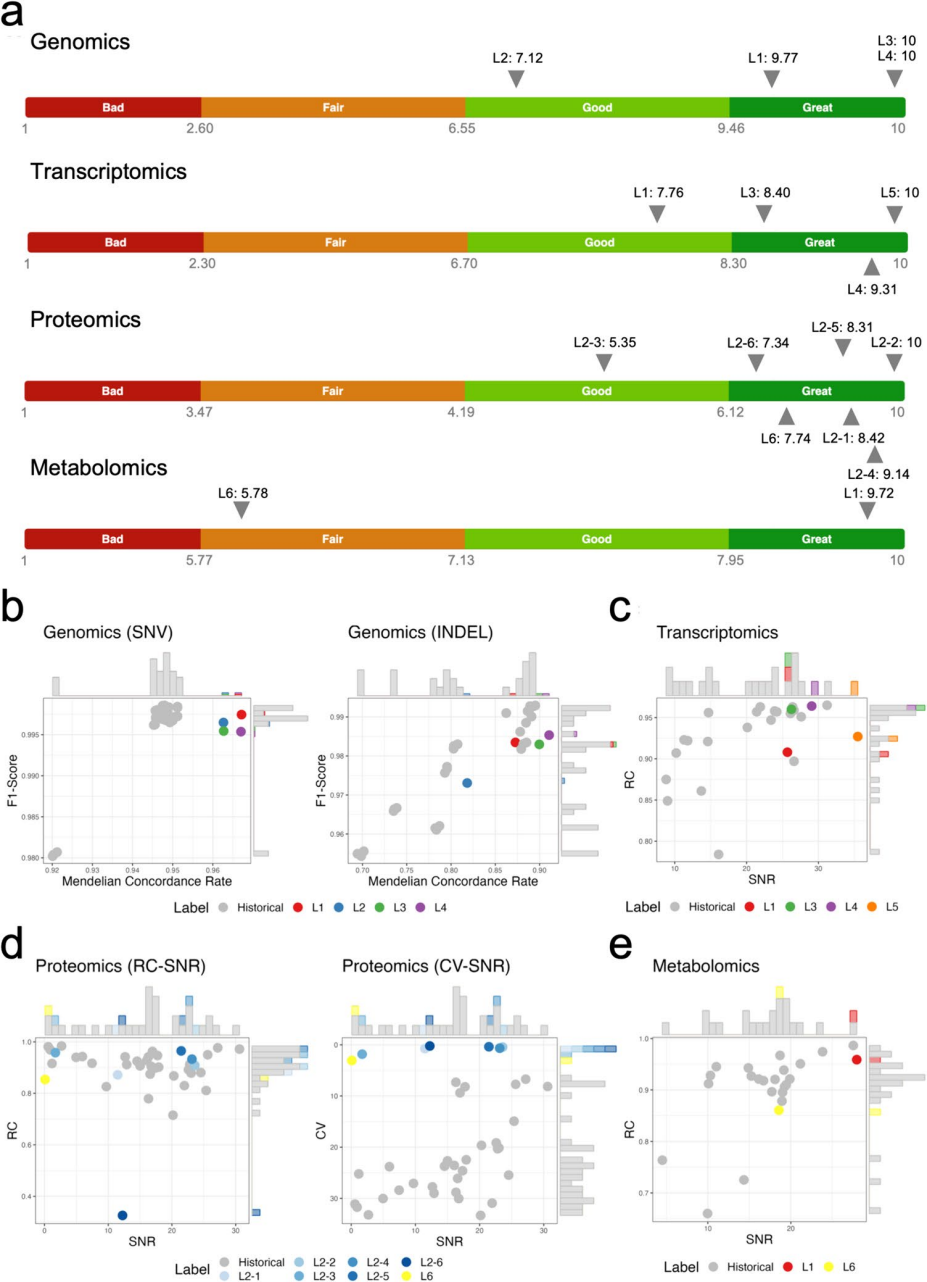
Quality assessment of multi-omics, multi-site, and multi-protocol datasets in proficiency testing. Chinese Quartet reference material was subjected to omics analysis in different EATRIS-Plus facilities. **a** The total quality scores of all datasets with ranking labels among all historical datasets in genomics, transcriptomics, proteomics and metabolomics. The label “Bad”, “Fair”, “Good” or “Great” manifests as the dataset ranking below the lower 20%, the 50%, the upper 20%, or above the upper 20% quantiles of the historical datasets. **b**–**e** Scatter plots of quality assessment results in genomics (**b**), transcriptomics (**c**), proteomics (**d**) and metabolomics (**e**) data; each datapoint shows the values of specific QC metrics across the samples in each dataset. F1-Score is the harmonic mean of precision and recall for variant calling. Signal-to-noise ratio (SNR) is defined as the ratio of the power of a signal to the power of noise. RC, the relative correlation with reference datasets, was calculated based on the Pearson correlation coefficient between the relative expression levels of a dataset for a given pair of groups and the corresponding reference fold-change values. *CV* Coefficient of variation. All historical datasets are colored gray to be distinguished from the tested datasets. All scatter plots were added with frequency distribution bars. Table S1 provides information on key aspects of the workflows. Table S2 provides the coordinates of the data points

Quartet reference materials were dispatched to an EATRIS-Plus coordinator site of this activity, aliquoted and then distributed to the various EATRIS laboratories for processing and analysis. Raw data resulting from these analyses were uploaded onto the Quartet Data Portal (https://chinese-quartet.org), where automated data analysis and reporting were conducted using publicly available workflows. For whole genome sequencing for example, the quality assessment starts from FASTQ files and can be divided into pre-alignment quality assessment, post-alignment assessment, and small variants calling results assessment. The quality of pre-alignment is assessed by FastQC and FastQ Screen, while post-alignment is assessed by Qualimap (http://qualimap.conesalab.org). The performance of variants calling results are evaluated by comparison to historic Fudan reference datasets, which had been provided by multiple laboratories, and Quartet family-dependent built-in genetic truth. MultiQC is used for compiling quality control (QC) results (for more details on the analysis pipelines, including the codes, please see https://docs.chinese-quartet.org/data_pipelines/intro/). EATRIS-Plus laboratories provided data for proteomics, metabolomics, DNA-seq and RNA-seq (Fig. 4). Three EATRIS sites also performed microRNA-seq, which was manually analyzed by the Fudan Quartet team, as this workflow was not implemented in the Quartet Data Portal at the time this study was performed (data no shown). Finally, one EATRIS-Plus site performed and evaluated microRNA qRT-PCR for 170 microRNAs (data not shown).

### Proficiency Testing of EATRIS-Plus Facilities

The Quartet design provides both reference dataset-dependent and -independent QC metrics for quality assessment of multi-omics profiling (Fig. 4). For qualitative omics, the F1-score is a commonly used measure that considers both false positives and false negatives by computing a harmonic mean of precision and recall. Quality metrics for assessing reliability of DNA-seq, RNA-seq, proteomics, and metabolomics in terms of intra-batch proficiency and cross-batch reproducibility are assessed using ratio-based reference datasets (Zheng et al. 2023). The Pearson correlation coefficient between the ratio-based expression levels of test datasets and reference datasets are used to describe the accuracy of quantitation. Signal-to-Noise Ratio (SNR) is used to investigate the differences between “cases” and controls. Overall, the platform allows the evaluation of the quality of data coming from different participants, platforms, protocols, and analytical tools.

One potential use of the Fudan Quartet reference samples is demonstrated here for liquid chromatography-mass spectroscopy (LC–MS) proteomics analysis. Laboratory L2 evaluated six different LC–MS proteomics protocols, being different in liquid chromatography column (performance (L2-2/4/6) versus endurance (L2-1/3/5)), MS mode (DDA (L2-1/2/5/6) versus DIA (L2-3/4)) and corresponding data analysis methods (for DIA: PaSER DIA-NN (L2-3/4), for DDA: MSFragger (L2-5/6) versus PaSER ProLuCID (L2-1/2) (Demichev et al. 2020; Meier et al. 2020; Meier et al. 2018; Xu et al. 2015; Yu et al. 2020). The quality of the proteomics output of these six protocols was assessed using the Fudan quality scoring yielding informative ranking that was subsequently used by the laboratory in their fit-for-purpose selection of workflows.

### The EATRIS-Plus Multi-omics Dataset

Implementation of FAIR principles (Wilkinson et al. 2016) at the individual omics level is essential to derive reproducible results from multi-omics data analyses. Most importantly, samples and data should be described by rich metadata. Since acquired data and subsequent multi-omics analysis can be affected by technical factors related to sampling, processing, sample storage conditions (temperature, duration, thawing/freezing cycles), and measurement conditions (protocols, measurement order, possible measurement batches), documentation of these factors can be key to reproducibility of research results. Additionally, intra- and inter-batch QC samples can help identify and adjust for batch effects (Cuklina et al. 2021). Finally, practices implementing FAIR Principles for Research Software can increase reproducibility of integrative multi-omics analyses (Barker et al. 2022; Chue Hong et al. 2022; de Visser et al. 2023).

The available analysis methods for multi-omics integration can be limited by type and completeness of data. While some integrative multi-omics methods can handle missing observations in some data modalities (Argelaguet et al. 2020; Cuklina et al. 2021), many methods require complete observations (Flores et al. 2023). Obtaining complete data for vertical integration is more challenging than in single omics experiments. Although methods that allow imputation of a low number of missing values are available, the choice of imputation method should be guided by the mechanism that causes missing values, e.g. missing at random vs. not at random (Wei et al. 2018). In the presence of numerous missing values, imputation is not advised.

Multi-omics studies have a large potential for the uncovering of biomarker profiles that are relevant for disease diagnosis, prognosis, or prediction of the efficacy of medical interventions (e.g. Demir Karaman and Isik 2023; Li and Zhou 2022; Wang et al. 2023; Xiao et al. 2022). They also contribute to study the impact of lifestyle on wellbeing on the molecular levels (Marabita et al. 2022), and the stratification of patients in clinical trials (Bourgonje et al. 2023; Zielinski et al. 2021). However, these biomarker profiles are subject to many different sources of technical and inter- and intraindividual variation. Capturing biological variability in healthy individuals and reducing the unwanted technical variation can help to plan and interpret future translational studies (Gallego-Pauls et al. 2021; Olshansky et al. 2022). To address these issues, EATRIS-Plus project executed a multi–omics study in a human population cohort of 127 individuals from the Czech Republic (ClinicalTrials.gov, Identifier: NCT04427163). These samples have been selected from a larger cohort of blood donor’s bio-banked at the Institute of Molecular and Translational Medicine (Palacký University Olomouc) to ensure sex balance and a representative distribution within the age range of 21 to 61 years. Blood samples were profiled with twelve different-omics technologies, genomics (whole genome sequencing (WGS) and array Comparative Genomic Hybridization (arrayCGH)), transcriptomics (mRNA and miRNA sequencing), and epigenomics (DNA methylation sequencing using EM-seq) on blood cells, and shotgun proteomics, targeted metabolomics (amino acids, very long chain fatty acids and acylcarnitines), untargeted lipidomics (positive and negative ion mode), and qPCR-based miRNA profiling on heparin and EDTA plasma, making this study one of the most comprehensive multi-omics studies executed until today. In this reference study, we applied various methods to uncover sources of technical and biological variation. To enable metadata harmonization and potential data integration with external studies, the Observational Medical Outcomes Data Model, OMOP, (https://www.ohdsi.org/data-standardization), was applied. This study serves to help design future multi-omics studies in human cohorts and help researchers choose the most appropriate omics layers, account for biological and technical confounders, calculate sample sizes, and creating robust experimental and computational workflows. The data from this study are currently prepared for publication and dissemination via local installation of cBioPortal to the community (https://cbioportal.imtm.cz). In addition, all standard operating procedures and data access information are provided in the Multi-omics Toolbox (MOTBX), which is described in the following section below.

### The EATRIS Multi-omics Toolbox (MOTBX)

The multi-omics research community still faces a number of challenges impacting the biomarker development and implementation in clinical practice that need to be overcome: (a) poor levels of technological, analytical and data processing harmonization resulting in poor reproducibility, (b) poor data stewardship and compliance to the FAIR principles (Wilkinson et al. 2016), (c) lack of understanding of the relationship between biomarkers belonging to different biological layers (transcriptomic, proteomic, metabolomic, epigenomic), (d) lack of reliable control reference values for these biomarkers, and (e) poor understanding of the actual clinical needs, resulting in limited clinical adoption (Taube et al. 2009).

In addition, information on omics reference material and multi-omics data integrative analysis and interpretation is fragmented in the knowledge space (Conesa and Beck 2019), and publicly available multi-omics profiling data are scarce.

Tackling these issues in a systematic way was one of the main objectives of the EATRIS-Plus project. This resulted in the development of the web based MOTBX (https://motbx.eatris.eu, https://zenodo.org/records/10141670).

**Fig. 5:**
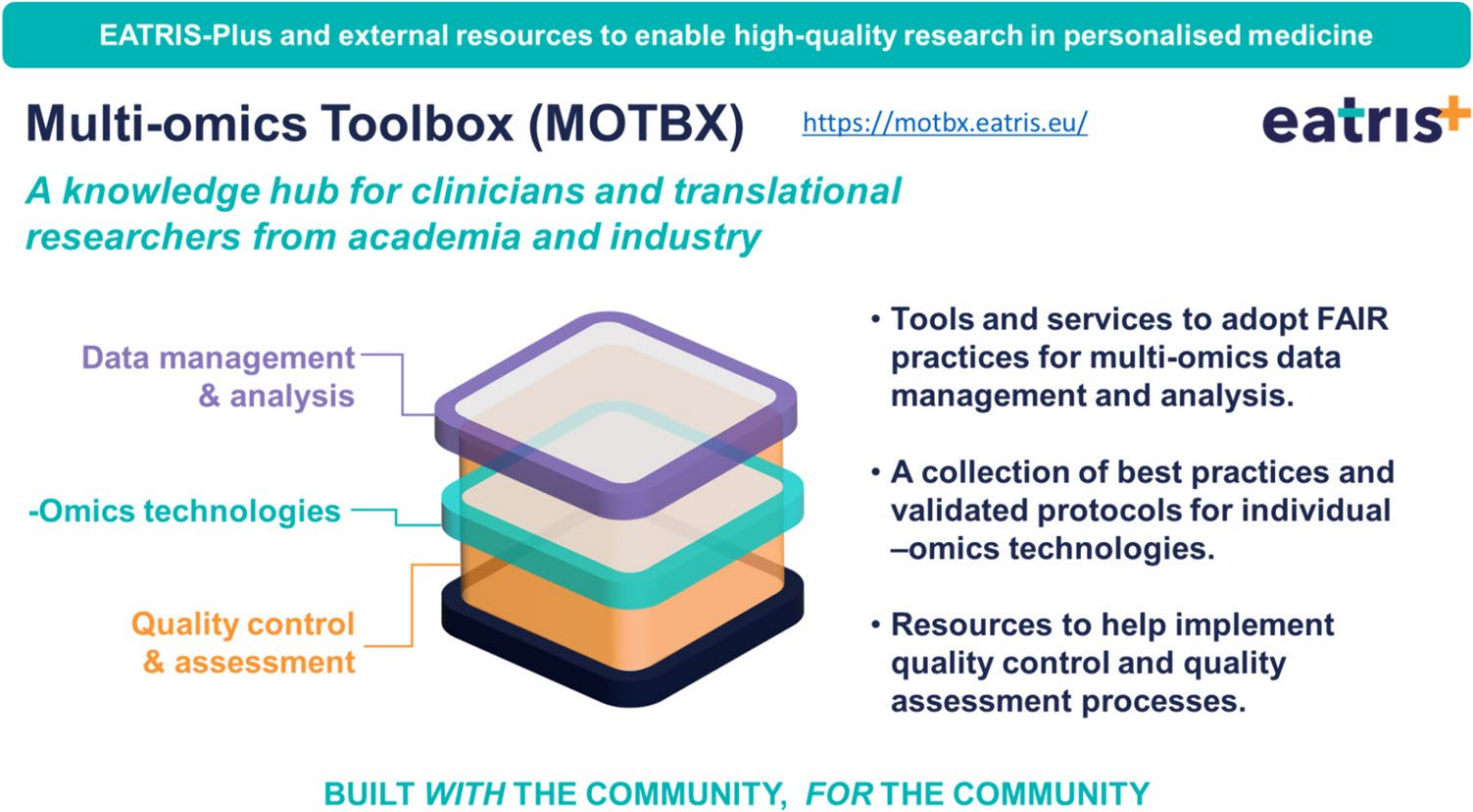
The multi-omics toolbox (MOTBX). The MOTBX  (https://motbx.eatris.eu) is an open access knowledge hub for translational researchers supporting development, implementation and adoption of multi-omics approaches for personalized medicine, including quality assessment aspects. The MOTBX has been developed by EATRIS-Plus partners across Europe to support the translational biomedical research communities, as the result of collaborative work on data stewardship, the generation of a multi-omics dataset of healthy individuals, quality assessment studies, and the help and input of stakeholders from industry and academia

The MOTBX is an open platform that is aimed to provide researchers, health professionals and other users with relevant information on resources related to multiple -omics technologies (genomics, epigenomics, transcriptomics, proteomics, and metabolomics), quality control and assessment, as well as data stewardship and integration. The toolbox core is structured into three sections: Omics Technologies, Quality Assessment, and Data, including analysis and FAIRification pipelines and tools (Fig. 5). It offers access to a collection of best practices and protocols for individual –omics technologies, resources to help implement quality control and quality assessment processes, tools and services to adopt FAIR data practices for multi-omics data management and analysis, education and training resources in multi-omics field, and inks to other EATRIS Toolkits, e.g., Patient Engagement Resource Centre (PERC), Innovation Management Toolbox (EJPRD IMT). The MOTBX is a resource that has been built with, and for the community. This means that researchers are not only welcome to use the MOTBX, but to support its further development by actively contributing with valuable resources. By providing such a toolbox to the research community, EATRIS aims to enable and support high-quality multi-omics research and accelerate the implementation of PM solutions.

## Discussion

Detection of sensitive, specific, and robust biomarkers is one of the main pillars of personalized medicine. The detection of biomarkers through omics technologies can greatly impact decision-making in healthcare, contributing to the early detection of disease onset, allowing for timely intervention and treatment, can help tailor treatments to individual patients based on their genetics, disease subtype, or response to therapy, leading to more effective, targeted and less toxic treatments (Ahmed 2022; Olivier et al. 2019). Omics biomarkers can help predict a patient's likelihood of developing certain diseases, distinguish similar pathological entities (differential diagnosis), provide prognostic information about a patient’s likelihood to progress, or serve as prognostic markers of a patient’s response to specific treatments, enabling proactive healthcare management strategies (Ahmad et al. 2023). In addition, in drug development and clinical trials, biomarkers based on omics can be used to identify suitable candidates for clinical trials, patient stratification, monitoring treatment response, and estimating drug efficacy (Subbiah 2023).

Overall, integration of biomarker detection with omics technologies empowers clinicians to deliver more informed decisions, leading to improved patient outcomes and a shift towards precision medicine (Johnson et al. 2021; Wang and Wang 2023). In this context, the procedures for sample harvesting and processing, data integration with phenotypic traits, analysis, and interpretation of results play a key role in the final decision on action. Depending on the outcome, this linear path may become circular or branch, when it is decided that further samples are needed, and other investigations are required to get a deeper understanding of the subject matter. The process requires quality assessment and quality control measures at every step: technology platforms should be validated, data collection and management transparent, data format and entries harmonized and electronically accessible, and the entire process including the analysis process and decision making should be thoroughly documented (Ali et al. 2023). European Biomedical Research Infrastructures have been developed since the early second decade of the 2000s to play a leading role in developing, applying, and disseminating quality assurance guidelines in different areas along the translational trajectory. For instance, the European biobanking research infrastructure (BBMRI, www.bbmri-eric.eu) is addressing quality in human biological samples and sample storage; EuroBioimaging (www.eurobioimaging.eu) is providing image analysis solutions and imaging quality assessment guidelines; ELIXIR (www.elixir-europe.org) has been founded with the goal to develop and provide guidance for management and quality of life science data. While these and other infrastructures exhibit a focus on specific technological expertise areas in translational research, EATRIS provides an integrative platform to prevent siloing. At the cost of overlapping with individual, focused services of other infrastructures, EATRIS ensures services and quality provision along the entire translational trajectory (Fig. 1).

In this work we share some of the initiatives EATRIS sites have participated in within the EATRIS-Plus project. Our efforts support the notion that proficiency testing of sample processing technologies and laboratory performance can and should be done at different steps of the translational process, as quality increases the chance of developing meaningful molecular tools. Longitudinal proficiency testing can lead to an improvement of laboratory performance, as it provides continuous feedback to the researcher on the quality of the sample processing. Application of commercial or research reference material serves multiple purposes, from assessing the quality of the data generation process at a given time point, the development of the quality over time, and the possibility to merge batches of data into larger datasets by applying statistical measures.

To demonstrate the applicability of the quality assessment strategies in the field of multi-omics applications mentioned above, EATRIS-Plus generated a multi-omics dataset, consisting of twelve-omics platforms, i.e. genomics (whole genome sequencing (WGS) and array Comparative Genomic hybridization (arrayCGH)), transcriptomics (mRNA and miRNA sequencing), epigenomics (DNA methylation sequencing using EM-seq) on blood cells, shotgun proteomics, targeted metabolomics (amino acids, very long chain fatty acids and acylcarnitines), untargeted lipidomics (positive and negative ion mode), qPCR-based miRNA profiling on heparin and EDTA plasma from about 100 healthy individuals (Czech cohort). We applied quality control and data integration strategies to create a holistic molecular representation of the individuals in the study. Together with references to literature on quality aspects of multi-omics data, links to analysis platforms and reference material, among others, these multi-omics data are available via MOTBX (https://motbx.eatris.eu), an open access multi-omics data platform that was created within the EATRIS-Plus project. The small sample size used in this “demonstrator” does not allow us to define the obtained results as reference values for each omics in a healthy population. The EATRIS-Plus community aims to make the multi-omics dataset available for further growth by data integration with other datasets, thereby increasing its value for biomedical research and biomarker discovery. The MOTBX, as a key public tool delivered by the EATRIS-Plus community, is a live resource open to the entire research community that will be updated and implemented with new relevant content generated by qualified EATRIS partners, beyond the EATRIS-Plus project lifetime.

Our efforts demonstrate that quality assessment is beneficial and feasible at every step of the translational trajectory. Testing and discussing the experiment design and plan against expert opinions, as provided, e.g., by the EATRIS Health Technology Assessment (HTA) services, can help focus on the study goal and outcome; longitudinal proficiency testing serves to assess a decline or improvement in sample processing quality; unwanted technical variation can be assessed and possibly alleviated via the processing of reference material, e.g. in studies where multiple batches of samples have to be processed. Comparative quality assessment can help harmonize and improve procedures and analysis approaches. We trust that our findings will be beneficial for researchers in the field, as well contribute to discussions within and across organizations that aim to improve healthcare and lifestyle through large scale studies (e.g., the Netherlands x-omics consortium (www.x-omics.nl) or the Healthy Brain Initiative (www.healthybrainstudy.nl).

Since multi-omics approaches seek to discover interconnected signatures for sample classification and network identification between cross-omics features, QC metrics should be suitable for evaluating the performance of each omics type in terms of data generation and integration, and should be related to these two research objectives: (a) the integration of multi-omics information for more robust sample classifiers and, (b) the identification of multilayered interconnected molecular signatures are the major goals for multi-omics profiling. Therefore, accessible multi-omics quality reference materials paired with fit-for-purpose performance metrics are urgently needed (Beger et al. 2019; Salit and Woodcock 2021; Sene et al. 2017; Wang et al. 2014). The use of multi-omics material in research is one of the measures that can be taken, but the definition of appropriate reference materials for omics implementation in clinical practice is one of the most critical aspects for omics adoption in health care systems for personalized medicine.

## Conclusion

Translational biomedical academic and clinical research requires step-by-step quality assessment and assurance measures in order to warrant successful clinical implementation of the findings. The European Infrastructure for Translational Medicine, EATRIS, provides resources, technologies, and expertise to assist along the translational trajectory (www.EATRIS.eu). With the Multi-omics Toolbox (https://MOTBX.EATRIS.eu), which is a community-driven “live resource” that will be fed and improved by new protocols, workflow and other resources, the EATRIS-Plus project has delivered open-source tools for multi-omics data quality assessment.

## Supplementary Information

Below is the link to the electronic supplementary material.

Supplementary file1 (XLSX 11 KB)

Supplementary file2 (XLSX 20 KB)

## Data Availability

All data are being made available via the Multi-omics Toolbox (www.MOTBX.eatris.eu). Fudan Quartet Reference Material can be requested from the co-authors of the Fudan Quartet team via http://chinese-quartet.org/#/dashboard

## Abbreviations

BBMRI: European biobanking research infrastructure
CV: Coefficient of variation
DNA: Deoxyribonucleic acid
EATRIS: European infrastructure for translational medicine
EJPRD IMT: European joint programme rare diseases innovation management toolbox
EQA: External quality assessment
FAIR: Findable, accessible, interoperable, and reusable
FFPE: Formalin-fixed paraffin-embedded
HTA: Health technology assessment
IBBL: Integrated BioBank of Luxembourg
LC–MS: Liquid chromatography-mass spectroscopy
MOTBX: Multi-omics toolbox
OMOP: Observational medical outcomes data model
PC: Principal component
PT: Proficiency testing
QA: Quality assessment
qRT-PCR: Quantitative reverse transcription polymerase chain reaction
RC: Relative correlation
RNA: Ribonucleic acid
SNR: Signal-to-noise ratio
SOP: Standard operating procedure
